# Therapeutic and immune-regulation effects of *Scutellaria baicalensis* Georgi polysaccharide on pseudorabies in piglets

**DOI:** 10.3389/fvets.2024.1356819

**Published:** 2024-03-04

**Authors:** Xianghua Shu, Ying Zhang, Xue Zhang, Ying Zhang, Yue Shu, Yulei Wang, Zhihui Zhang, Chunlian Song

**Affiliations:** ^1^College of Veterinary Medicine of Yunnan Agricultural University, Kunming, China; ^2^The Faculty of Science and Mathematics, Auburn University, Auburn, AL, United States; ^3^The Faculty of Veterinary Medicine, Chiang Mai University, Chiang Mai, Thailand; ^4^Faculty of Veterinary Medicine, Khon Kaen University, Khon Kaen, Thailand

**Keywords:** piglets, therapeutic effect, immune-regulation effect, pseudorabies virus, *Scutellaria baicalensis* Georgi polysaccharide, *Rodgersia sambucifolia* Hemsl flavonoids

## Abstract

Pseudorabies virus (PRV) can cause fatal encephalitis in newborn pigs and escape the immune system. While there is currently no effective treatment for PRV, *Scutellaria baicalensis* Georgi polysaccharides (SGP) and *Rodgersia sambucifolia* Hemsl flavonoids (RHF) are traditional Chinese herbal medicines with potential preventive and therapeutic effects against PRV infection. In order to explore which one is more effective in the prevention and treatment of PRV infection in piglets. We investigate the therapeutic effects of RHF and SGP in PRV-infected piglets using clinical symptom and pathological injury scoring systems. The immune regulatory effects of RHF and SGP on T lymphocyte transformation rate, cytokines, T cells, and Toll-like receptors were also measured to examine the molecular mechanisms of these effects. The results showed that SGP significantly reduced clinical symptoms and pathological damage in the lungs, liver, spleen, and kidneys in PRV-infected piglets and the T lymphocyte conversion rate in the SGP group was significantly higher than that in the other treatment groups, this potential dose-dependent effect of SGP on T lymphocyte conversation. Serum immunoglobulin and cytokine levels in the SGP group fluctuated during the treatment period, with SGP treatment showing better therapeutic and immunomodulatory effects in PRV-infected piglets than RHF or the combined SGP + RHF treatment. In conclusion, RHF and SGP treatments alleviate the clinical symptoms of PRV infection in piglets, and the immunomodulatory effect of SGP treatment was better than that of the RHF and a combination of both treatments. This study provides evidence for SGP in controlling PRV infection in piglets.

## 1 Introduction

Pseudorabies virus (PRV) is extremely infectious in mammals and poses a risk of spill-over transmission to humans (1) and considerable public health challenges. PRV can inhibit host innate immunity and promote viral replication (2). Thus, focusing more on immunity regularity is important for investigating the effect of PRV. For humoral immunity, immunoglobulins IgG, IgA, and IgM are important for immunity against viral infections, with sIgA preventing the local invasion of the virus, and IgG and IgM blocking the spread of the virus through the blood by neutralizing and opsonizing viruses. For cellular immunity, The T lymphocytes have antiviral (3), antifungal, and immunomodulatory functions (4). The T lymphocyte transformation test is a definitive test for detecting the state of cellular immune function, where higher T lymphocyte conversion rates reflect a more pronounced role for T lymphocytes in enhanced immunity (5). Furthermore, CD4^+^ T cells coordinate the immune response and secrete different lymphocytes that act on the surrounding antigen-presenting cells (6), CD8^+^ T cells have killing and inhibitory functions that are crucial to the immune system (7); thus, the CD4^+^/CD8^+^ ratio can act as an indicator of immune capacity (8). There are also some differences among the different periods of the immune response, during the reactive phase, interleukin (IL)-2 and IL-4 promote the activation, proliferation, and differentiation of T and B cells (9), then, the practical phase, IFN-γ can activate macrophages, enhance their phagocytic and killing activities, and inhibit viral replication (10). In addition, Toll-like receptors (TLRs) possess antiviral biological activities and are significant components of the intrinsic immune system. TLR3 and TLR7 on endosomes recognize nucleic acids (11); TLR3 primarily recognizes double-stranded viral RNA, suggesting that it has important antiviral functions (12), while TLR7 recognizes single-stranded viral RNA. These TLRs are essential for protecting the body against infection (13). To sum up, this immunity helps us to evaluate the immunomodulatory effects of drugs.

*Scutellaria baicalensis* Georgi (SG) is also known as Chinese skullcap or Huangqin and is a perennial herb in the Lamiaceae Martinov (14). Recent clinical applications of Chinese skullcaps include the treatment of many diseases, including inflammation (15, 16), hypertension (17), cardiovascular disease (18), neurodegeneration (19), and tumors (20). Current studies have confirmed that polysaccharides have a wide range of therapeutic effects and disease-prevention properties, with most traditional Chinese medicinal polysaccharides acting as immune enhancers *in vitro* and *in vivo*. Moreover, the dried rhizome of *Rodgersia sambucifolia* Hemsl (RH), known as Yantuo, or Maoqinggang, is effective in clearing heat, detoxification, dispelling wind, and draining dampness; it has astringent properties and is often used to treat colds, headaches, rheumatism, bone pain, and traumatic bleeding (21). Flavonoids also have important immunomodulatory effects and can play antiviral and anti-infection roles. However, the effect of *Scutellaria baicalensis* Georgi polysaccharide (SGP) and *Rodgersia sambucifolia* Hemsl Flavonoid (RHF) on PRV has not been reported.

In order to screen out effective Chinese medicine against PRV, previous research from our group evaluated the safety and immune effects of extracts from RH, *Angelica*, SG, and the root of *Isatis* indigotica sourced from Yunnan. RHF and SGP with superior immunomodulatory effects were selected for this study to investigate the therapeutic and immunomodulatory effects of RHF and SGP on piglets infected with PRV.

## 2 Methods

### 2.1 Laboratory animals and virus sources

Fifty 15 ± 3 days-old Saba × Duroc hybrid pigs were selected from the Luquan Mountain area of Kunming City, Yunnan Province. Tests for porcine reproductive and respiratory syndrome (PRRSV), PRV, porcine circovirus type 2 (PCV2), and classical swine fever virus (CSFV) antigens were negative. All piglets were born from unvaccinated sows and tested negative for PRV, PCV2, PRRSV, CSFV, and porcine parvovirus using a polymerase chain reaction (PCR) method. The piglets were determined to be free of antibodies against PRV (gE) prior to the study using enzyme-linked immunosorbent assay (ELISA) kits. The piglets were reared under average daylight conditions and provided with standard commercial feed and unrestricted access to water. The PRV gD strain YN (22) was isolated from the lungs of infected pigs in Yunnan Province and used in this study. The PRV titre was 10^5.5^ TCID_50_/0.1 mL (PK-15 cell line).

### 2.2 Preparation of *Rodgersia sambucifolia* Hemsl flavonoids and *Scutellaria baicalensis* Georgi polysaccharide

#### 2.2.1 *Rodgersia sambucifolia* Hemsl flavonoids

Dried clover roots of *Rodgersia sambucifolia* Hemsl. were ground and soaked in 60% ethanol for 24 h at a solid–liquid ratio of 1:60. The mixture was sonicated for 15 min before being placed in a water bath at 70°C for 3 h. The ethanol was evaporated, and the residue was dried. The flavonoid content of the extract was 41.1%, which was determined using the method described previously (23).

#### 2.2.2 *Scutellaria baicalensis* Georgi polysaccharide

The roots of *Scutellaria baicalensis* was dried, crushed, and boiled in distilled water at a material-to-liquid ratio of 1:50 for 20 min. The residue was removed from the filtrate, which was then mixed with 95% ethanol and maintained at 4°C overnight. The supernatant was removed to obtain a flocculated precipitate, which was dried to a constant weight at 55°C to obtain the baicalin polysaccharides. The polysaccharide content was 40.3%, which was determined using a previously described method (24).

### 2.3 Lymphocyte proliferation assay

Peripheral lymphocytes were isolated from the PRV-and anti-PRV-antibody-free piglets as previously described (25). Trypan blue staining was used to perform cell counts from cells cultured in serum-free RPMI 1640 complete solution suspensions. We used a whole cell density of 5 × 10^9^ cells/mL in our experiments. A total of four experimental groups were used (I, II, III, and IV; Table 1) and each experimental group was repeated four times. Cells were incubated for 44 h under 5% CO_2_ before 5 μL MTT were added to each well under dark conditions. After a 4 h incubation, the absorbance at 570 nm was measured with a UV spectrophotometer. The experiment was repeated three times.

**Table 1:** Experimental grouping.

Groups	Treatments
Group I	Cell suspension at different concentrations (1.25–5 mg/mL)
Group II	Cell suspension
Group III	RPMI 1640 complete culture medium at different concentrations (1.25–5 mg/mL)
Group IV	RPMI 1640 complete culture medium

The stimulus index (SI) was calculated as follows (26):


$$SI=\left\{\left(OD_{I}-OD_{III}\right)/\left(OD_{II}-OD_{IV}\right)\right\}\times 100\%$$

### 2.4 Piglet PRV infection experimental design

After 1 week of domestication, 50 piglets were randomly divided into five groups with 10 piglets in each group. The first group was the control, in which piglets were administered 1 mL saline solution intramuscularly. The second group included PRV-infected piglets (PRV), and the third and fourth groups were composed of PRV-infected piglets treated with either 200 mg/kg RHF (PRV + RHF) or 200 mg/kg SGP (PRV + SGP), respectively. The fifth group included PRV-infected piglets treated with 100 mg/kg RHF and 100 mg/kg SGP (PRV + RHF + RHF).

On day 0, piglets in groups 2–5 were administered 1 mL PRV nasal drops and the control group received PBS. After infection, groups 3, 4, and 5 received intranasal administration (i.g.) of the corresponding treatments once a day for 21 consecutive days, while distilled water was intragastrically administered to piglets in groups 1 and 2.

#### 2.4.1 Animal ethics statement

All experiments performed in this study were approved by the International Animal Care and Use Committee of the Yunnan Agricultural University (permission code: YAUACUC06; date of publication: July 10, 2017). The study complied with the guidelines of the institutional administrative and ethics committees for laboratory animals.

### 2.5 Therapeutic effect

#### 2.5.1 Evaluation of clinical symptoms

Either 1 mL PRV or PBS was intranasally administered to each piglet and clinical symptoms were recorded and evaluated daily (Table 2).

**Table 2:** Evaluation of clinical symptoms.

	Grade^*^	Symptoms
Clinical evaluation	0	No general clinical or neurological signs
	1	Cough, shortness of breath, rough coat, and decreased food consumption
	2	Inactive, slow moving, and dyspnoea
	3	Diarrhoea, shaky movements, incoordination, wall scraping, tremors, or limb weakness
	4	Inability to stand, limb paralysis, moribund state, or death

The highest of the two scores on each day was recorded as the individual daily score.

#### 2.5.2 Evaluation of pathological injury

At 14 days post-infection (dpi), the piglets were euthanized with 100 mg/kg pentobarbital sodium intravenous injection and the liver, spleen, lung, and kidney tissues were collected and preserved in 10% paraformaldehyde. Each tissue sample was cut into 5 mm × 5 mm × 3 mm sections and processed following standard procedures. Formalin-fixed, paraffin-embedded tissue (FFPE) sections 5 μm in thickness were stained with haematoxylin and eosin (H&E) for microscopy (Motic China Group Co., Ltd.). Lung injury was scored using the Smith score (27) (Table 3), liver injury was scored using the Suzuki pathological score (28) (Table 4), and spleen injury was assessed using a scoring system for the pathological changes observed in PRV-infected piglets (Table 5). Renal injury was evaluated using the Haas grading system (Table 6).

**Table 3:** Lung injury score.

Score	Inflammation	Bleeding	Hyaline membrane formation
0	None	None	None
1	Slight	Slight	Single cell
2	Mild	Mild	<30%
3	Moderate	Moderate	31–60%
4	Severe	Severe	>60%

The total lung injury score was calculated as the sum of the clinical symptoms. Each sample was observed under 400× magnification, and the average values were recorded.

**Table 4:** Liver injury score.

Score	Congestion	Vacuole degeneration	Necrosis
0	None	None	None
1	Slight	Slight	Single cell
2	Mild	Mild	<30%
3	Moderate	Moderate	3–60%
4	Severe	Severe	>60%

The total liver injury score was calculated as the sum of the individual scores for each symptom. Each sample was observed under 400× magnification, and the average values were recorded.

**Table 5:** Spleen injury score.

Score	Inflammatory cell infiltration	Bleeding	Plasmacytosis
0	None	None	None
1	Slight	Slight	Single cell
2	Mild	Mild	<30%
3	Moderate	Moderate	31–60%
4	Severe	Severe	>60%

The total splenic injury score was calculated as the sum of the individual scores for each symptom. Each sample was observed under 400× magnification, and the average values were recorded.

**Table 6:** Kidney injury score.

Score	Inflammatory cell infiltration	Bleeding	The integrity of the glomerular structure
0	None	None	>90%
1	Slight	Slight	>60%
2	Mild	Mild	31–60%
3	Moderate	Moderate	<30%
4	Severe	Severe	None

The total kidney injury score was calculated from the sum of the score for each individual symptom. Each sample was observed under 400× magnification, and the average values were recorded.

### 2.6 Immune-regulation effect

#### 2.6.1 Enzyme-linked immunosorbent assay

A specific enzyme-linked immunosorbent assay (ELISA) kit (Shanghai Enzyme-linked Biotechnology Co., Ltd., China) was used to detect the serum levels of IgA, IgM, IgG, IL-2, IL-4, IFN-γ, CD4^+^, CD8^+^, and sIgA antibodies following the manufacturer’s instructions.

#### 2.6.2 Quantitative reverse transcriptase PCR

Total RNA was isolated from spleen tissue using TRIzol reagent (Takara, China) and cDNA was synthesised using the First-Strand cDNA Synthesis Kit (Takara, China). Quantitative reverse transcription PCR (qRT-PCR) was performed using a one-step SYBR PrimeScriptTM RT-PCR Kit II (Takara Biotech Co., Ltd., China). *β-actin* was used as a reference gene to detect *TLR3* and *TLR7* expression and quantified using the 2^(−ΔΔCt)^ method for statistical analysis. Each assay was repeated three times. The primers used in this study were as follows:

*β-actin*: (F) TCTGGCACCACACCTTCT, (R) TGATCTGGGTCATCTTCTCAC

*TLR3*: (F) TCCAACTAACAAACCAGGC, (R) ACATCCTTCCACCATCT

*TLR7*: (F) TGCTTTCCAGTTGCGACATC, (R) CAGACAAGCCACACAGCGTC.

### 2.7 Statistical analysis

Data are expressed as the mean ± standard deviation. A one-way analysis of variance (ANOVA) was performed, followed by the Student–Newman–Keul multiple comparison test to compare the means. A *p*-value <0.01 was considered statistically significant. Analyses were performed using the SYSTAT 9 software package (SPSS 20).

## 3 Results

### 3.1 RHF and SGP relieve the clinical symptoms of pseudorabies

**Figure 1:**
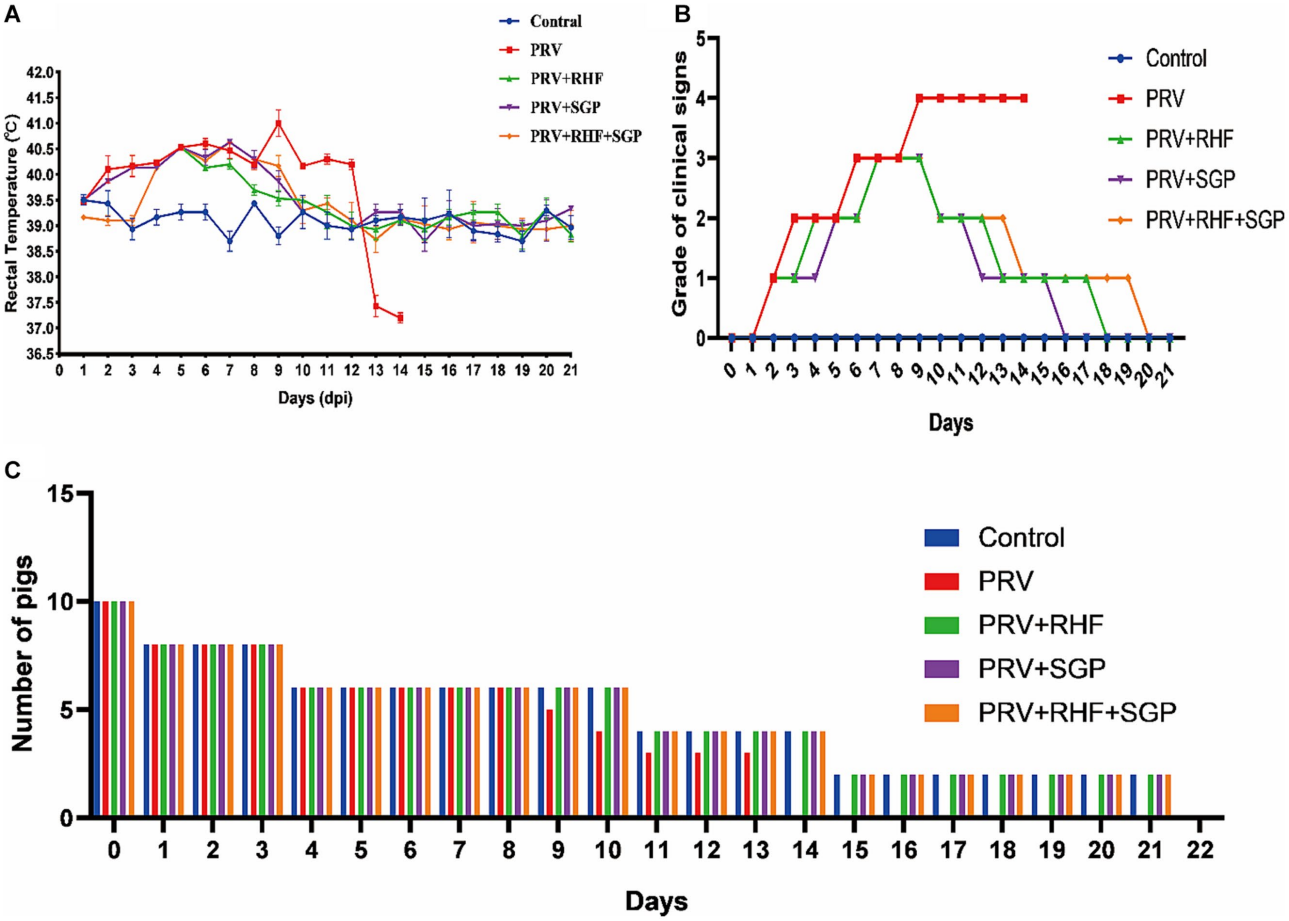
The clinical signs of pseudorabies virus-infected piglets. **(A)** The rectal temperature change of each group. **(B)** Clinical signs grade of piglets in each group. **(C)** The number of piglets surviving in each group.

After 3 days post infection (dpi), the rectal temperature of the pigs in the four PRV-infected groups was higher than that of the control group (*p* ≤ 0.01). After 9 dpi, the rectal temperature decreased in the treated group (*p* ≤ 0.05) before it stabilised after 10 dpi (*p* > 0.05), whereas that in the PRV group remained higher than that of the control (*p* ≤ 0.01) (Figure 1A). At 14 dpi, all piglets in the PRV group died, with a mortality rate of 100%, while those in the treatment groups 60% (6/10) (Figure 1C). These results suggested that RHF and SGP alleviated fever symptoms in piglets after PRV infection, with no significant difference between the treatment conditions.

At 9 dpi, the piglets in the PRV group were inability to stand, exhibited limb paralysis, and finally died at 14 dpi. In the RHF group, the clinical symptom scores initially decreased to 2 points 10 dpi and returned to normal 13 and 18 dpi. The score in the RHF + SGP group decreased to 2 points at 13 dpi, then to 1 point at 14 dpi before it returned to normal at 20 dpi. In contrast, the scores in the SGP group dropped to 1 at 12 dpi and then returned to normal at 16 dpi (Figure 1B). Overall, the clinical symptoms of the PRV-infected piglets were alleviated in all three drug groups, with the greatest effect observed in the SGP group when compared to that in the RHF or RHF + SGP treatment groups.

### 3.2 RHF and SGP treatments reduce pathological injury in PRV-infected piglets

**Figure 2:**
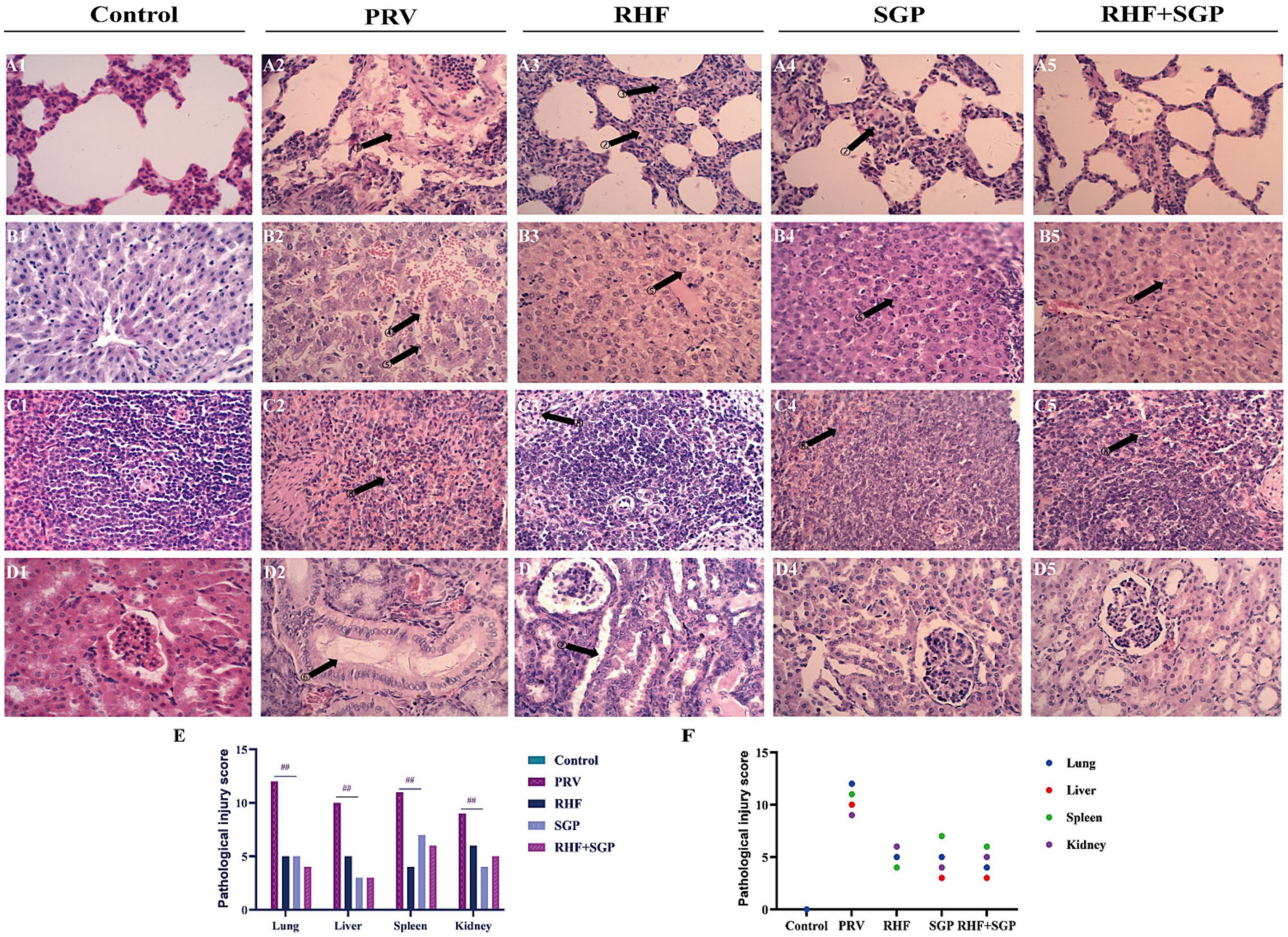
Histopathological pictures of lung, liver, spleen, and kidney tissues in different experimental groups and the pathological injury score. **(A)** Histopathological pictures of the lung. **(B)** Histopathological pictures of the liver. **(C)** Histopathological pictures of the spleen. **(D)** Histopathological pictures of the kidney. **(E)** The pathological injury scores. **(F)** Correlation between pathological damage and injury score within the same tissue. The arrow points to the lesion site. The numbers above the arrows indicate serous exudation in the alveolar interstitium; alveolar wall thickening; increased red blood cells; hepatocyte cord disorder, hepatic sinusoid enlargement; abundant plasma cells; distal convoluted tubule structure changes; the structure of distal convoluted tubules and proximal convoluted tubules are fuzzy; lymphocyte infiltration and erythrocytosis. (Hematoxylin and eosin staining, 400× magnification). (*n* = 10 in per group).

The H&E-stained lung tissue sections from the PRV group showed focal bleeding, alveolar stenosis, serous exudation, tumour inflammatory cell infiltration, and epithelial cell shedding when compared with those from the control group. The liver tissue sections from the PRV group showed a disordered arrangement of the hepatocyte cords, severe haemorrhage between hepatocytes, hepatocyte necrosis, nucleolar vacuoles, and widening of the hepatic sinuses; while the spleen sections showed a scattered structure in the germinal centre, partial necrosis of lymphocytes, serous exudate in the interstitial tissue, and increased red myeloid erythrocytes; and the kidney sections showed inflammatory erythrocytes in the glomerulus and kidney capsule, shedding of the epithelial cells in the renal tubules, and structures of the glomerulus, distal tubules, and proximal tubules were disordered. In the treatment groups, lung exudation and bleeding were reduced, liver tissue bleeding was reduced, the hepatic cord was neatly arranged, the spleen structure was intact, the germinal centre was dense, and the kidney structure was intact. Together, these results showed that both RHF and SGP alleviated the pathological damage to the heart, liver, spleen, lungs, and kidneys of PRV-infected piglets (Figures 2A–D).

Pathological damage scores showed that damage to the lungs, liver, spleen, and kidneys of PRV-infected piglets was alleviated in the three drug groups when compared to those of the untreated control groups (*p* ≤ 0.01) (Figure 2E), and the pathological lesions correlated with damage scores within the same tissue (Figure 2F).

### 3.3 SGP effectively enhances T lymphocyte conversion rates

**Figure 3:**
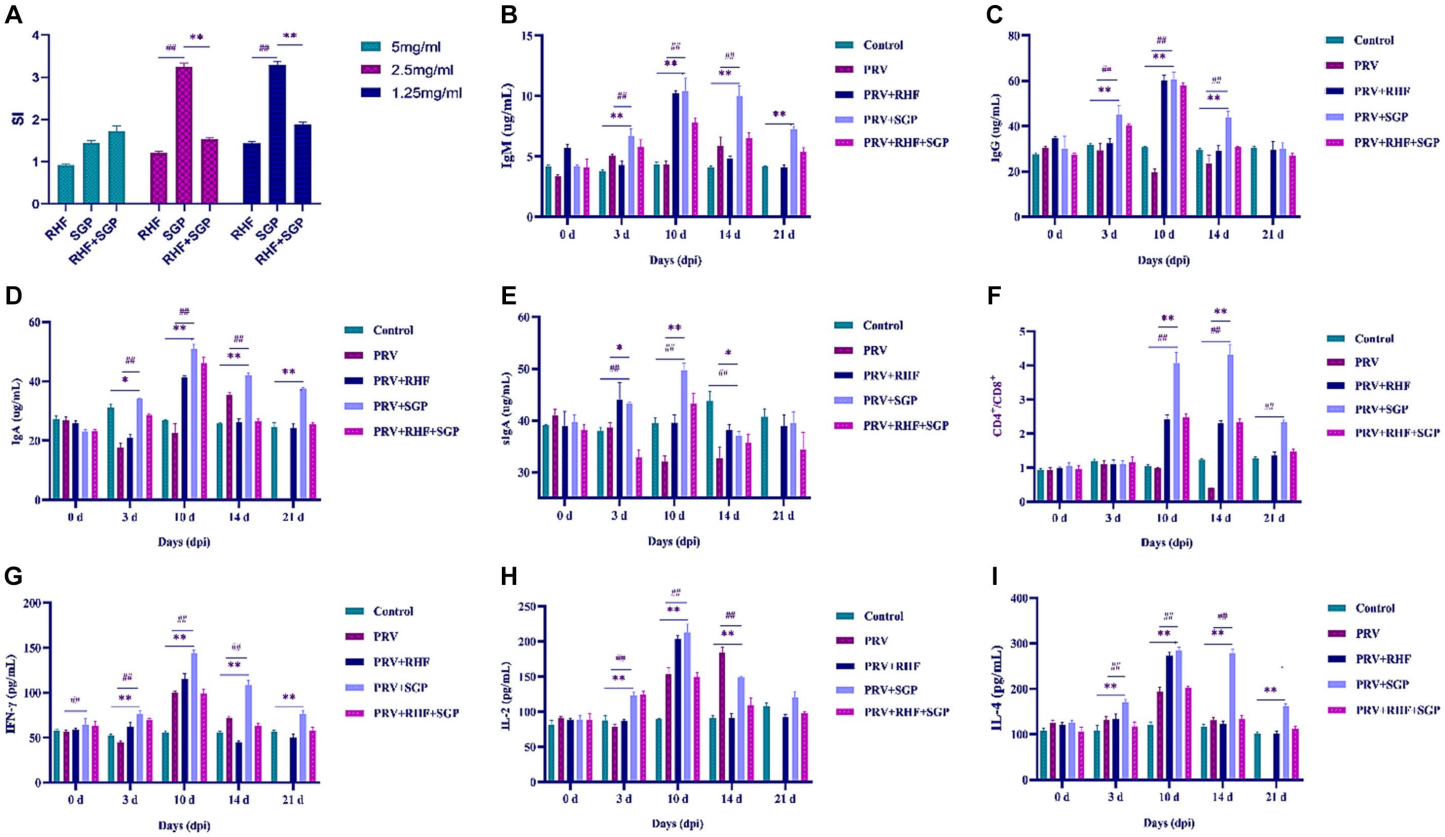
**(A)** The stimulation index in each group. **(B)** IgM level in each group. **(C)** IgG level in each group. **(D)** IgA level in each group. **(E)** sIgA level in each group. **(F)** CD4^+^/CD8^+^ ratio in each group. **(G)** IFN-γ level in each group. **(H)** IL-2 level in each group. **(I)** IL-4 level in each group. (At each selected time point, all the living piglets were tested, *n* = 10 in per group).

The effect of SGP and RHF on T lymphocyte conversion was investigated in PRV-infected piglets. The stimulation index (SI) of 1.25 mg/mL and 2.5 mg/mL SGP treatments was significantly higher than that of 5 mg/mL concentrations in PRV-infected piglets (*p* ≤ 0.01) (Figure 3A). These results indicated that SGP effectively enhances T lymphocyte conversion rates.

### 3.4 RHF and SGP treatments increase immunoglobulin concentration

The levels of IgM, IgG, IgA, and sIgA in the experimental groups were significantly different from those at 3 dpi (*p* ≤ 0.01 or *p* ≤ 0.05). These differences were greatest at 10 dpi (*p* ≤ 0.01), after which IgM, IgG, and IgA decreased, while sIgA first decreased and then increased when compared with control group (Figures 3B–E). Both RHF and SGP increased the concentration of immunoglobulins in the blood and lung tissue, with SGP showing a greater ability to boost immunoglobulin levels, indicating an improved immunity and mucosal anti-infection immunity in piglets infected with PRV.

### 3.5 SGP improves the serum CD4^+^/CD8^+^ ratio and cytokine production in PRV-infected piglets

Significant differences in the CD4^+^/CD8^+^ ratio in the SGP group were observed at 10 dpi when compared with control group (*p* ≤ 0.01) (Figure 3F). Significant differences in the levels of IFN-γ, IL-2, and IL-4 in all experimental groups were observed at 3 dpi, with the largest differences observed at 10 dpi when compared with control group (*p* ≤ 0.01). These results showed that SGP improved the serum CD4^+^/CD8^+^ ratio and cytokine production in PRV-infected piglets (Figures 3G–I).

### 3.6 SGP increases *TLR3* and *TLR7* expression in PRV-infected piglet spleens

**Figure 4:**
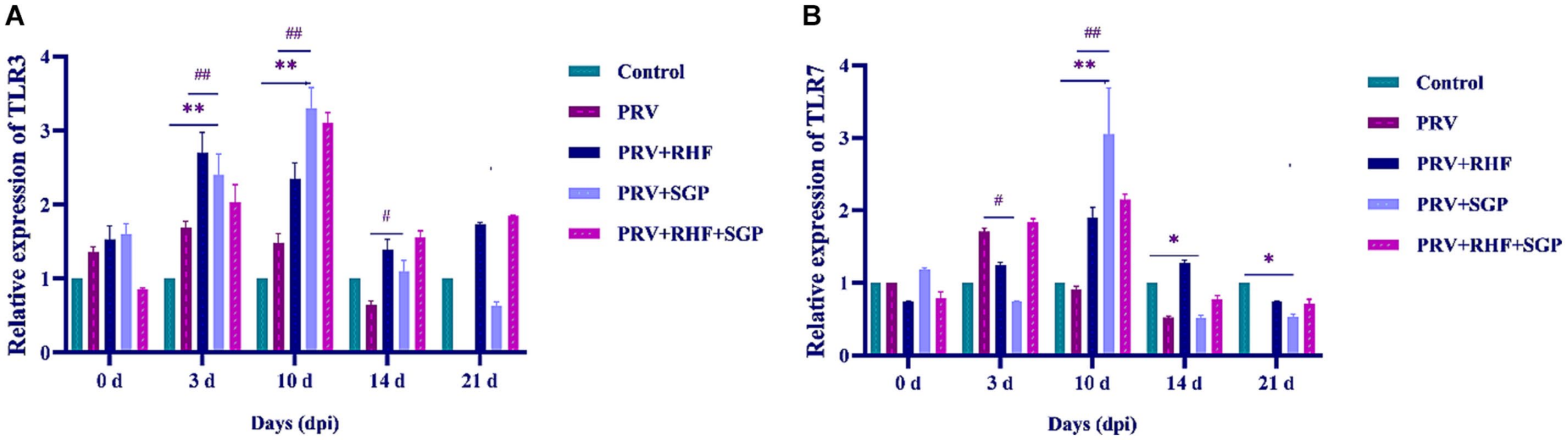
**(A)** TLR3 level in each group. **(B)** TLR7 level in each group. (*n* = 10 in per group).

A significant increase in the *TLR3* mRNA levels in PRV-infected piglets treated with and without SGP and RHF were observed at 3 dpi (*p* ≤ 0.01), with the largest increase observed at 10 dpi when compared with the controls (*p* ≤ 0.01). These increases were followed by a gradual decrease in *TLR3* levels. The *TLR7* levels first decreased, then increased, and then decreased again after SGP and RHF treatment, with the highest expression observed at 10 dpi when compared with the controls. Both RHF and SGP treatment increased the relative expression levels of *TLR3* and *TLR7* in the spleens of PRV-infected piglets; however, this effect was more significant in the SGP group (Figures 4A,B).

## 4 Discussion

Several studies have shown that traditional Chinese medicines have antiviral and immune-enhancing effects (29). RH is used to treat various immune diseases, such as tumours, asthma, and rheumatoid arthritis (21), although the medicinal value of these active ingredients has rarely been reported (30). SG is the dried *S. scutellariae* root from which baicalin is extracted from the by-product of *Scutellaria* flavones. These compounds are relatively easy to obtain and demonstrate anti-tumour activities, blood glucose regulatory effects, lipid regulation, antioxidant properties, antibacterial activates, and anti-inflammatory and immunomodulatory effects (31, 32). The prevention and treatment of PRV pose considerable challenges to industrial pig farming and the effects of RSH and SGP on PRV-infected piglets have not been reported (see Figure 4).

We initially fermented and extracted the effective components from four native Yunnan herbs (SG, RH, *Angelica*, and the root of *Isatis* indigotica) and found that SG and RH were the most effective medicinal herbs. We sought to identify the medicinal herb with optimal therapeutic and immunomodulatory effects on PRV-infected piglets and determine whether their individual or combined administration is most effective at treating PRV infection. Clinical symptom and pathological injury scores were used to evaluate the therapeutic effects of the experimental treatments in PRV-infected piglets and lymphocyte transformation assays and relevant immunoglobulins, cytokines, T cells, and Toll-like receptors were evaluated to demonstrate the immunomodulatory effects of the tested treatments.

The clinical symptom and pathological injury scores showed that all three treatment groups alleviated the clinical symptoms and pathological injury of pseudorabies in PRV-infected piglets. Xiong et al. (33) calculated the clinical cure rate of COVID-19 patients using a Chinese herbal formula containing *Scutellaria* and found that the clinical and fever symptom scores and inflammatory biomarkers were reduced. Ming et al. (21) also found that *Astragalus* polysaccharides alleviated lipopolysaccharide-induced inflammatory lung injury by altering the intestinal microbiota in mice, while Li et al. (31) reported that the flavonoid compound, kaverol, inhibits PRV replication in the brain, lungs, kidneys, heart, and spleen, alleviating pathological changes in these organs.

The levels of IgM, IgG, IgA, sIgA, IL-2, IL-4, TLR3, and TLR7 showed that both RHF and SGP enhanced the immunity of PRV-infected piglets; however, SGP had a stronger effect than RHF. In lymphocyte transformation experiments, we found that 1.25 mg/mL and 2.5 mg/mL SGP treatments could improve lymphocyte conversion, indicated by the increased immunoglobulin concentration in piglets that received SGP treatment than that of untreated piglets. Baicalin can significantly increase IgA, IgG, and IgM levels in the serum of mice (23), which is similar to the results of the present study. Pro-inflammatory cytokines and high levels of interferon in the airway mucosa can trigger and maintain local inflammation and can lead to disturbances in the protective mucosal immune response. We found that sIgA levels in treated piglets at 21 dpi increased instead of decreased. We hypothesized that this was related to higher levels of interferon that aggravated the mucosal inflammatory response (26, 34). Both RHF and SGP increased the ratio of CD4^+^/CD8^+^ cells and cytokine levels in the serum of PRV-infected piglets, although SGP was more effective. Future studies will examine the relationship between interferon and PRV infection.

The SGP treatment of PRV-infected piglets showed a greater increase in the relative RNA expression of *TLR3* and *TLR7* in isolated spleen tissues, further indicating that RHF and SGP treatment improved the immunomodulatory function in PRV-infected piglets and that the effect of SGP treatment was greater than that of RHF. There are also some current articles suggesting that the rise of TLR4 in herbal formulations containing baicalin is also associated with inflammatory bursts (35).

Most markers for inflammation showed higher elevation at 10 dpi, which may be related to the immune response against PRV. Previous study have shown that PRV has the highest viral load between 7 and 10 dpi and that antibody detection in the humoral immune response after immunisation occurs after 5 to 10 dpi (36). Therefore, we hypothesised that administration of RHF and SGP within 10 dpi of infection would increase the levels of PRV-related immune factors in piglets, promote lymphocyte proliferation, and enhance piglet resistance to PRV. Our results showed that RHF and SGP can reduce the clinical symptoms and pathological damage in PRV-infected piglets by improving their immunity.

In this study, the *in vitro* tests had some limitations and in the future, primary cells can be isolated from challenged tissue and transcriptomic analysis can be used to search for proteins with significant differences. Validation was performed on primary cells and the safety of these two extracts on cells needs further investigation. Network pharmacological analysis of the baicalin monomer and PRV will be conducted in the future to find targets and functional pathways that will provide information to produce more effective drugs suitable for treating PRV.

## 5 Conclusion

RHF and SGP treatments alleviate the clinical symptoms of PRV infection in piglets. The immunomodulatory effect of SGP treatment was better than that in the RHF and a combination of both treatments. This study has important implications in the search for effective herbal medicines that can control PRV transmission and infection.

## Data availability statement

The original contributions presented in the study are included in the article/supplementary material, further inquiries can be directed to the corresponding author.

## Ethics statement

The animal study was approved by International Animal Care and Use Committee of the Yunnan Agricultural University (permission code: YAUACUC06; date of publication: July 10, 2017). The study was conducted in accordance with the local legislation and institutional requirements.

## Author contributions

XS: Writing – review & editing. YZ (2nd author): Writing – original draft. XZ: Visualization, Writing – review & editing. YZ (4th author): Methodology, Writing – review & editing. YS: Conceptualization, Writing – review & editing. YW: Data curation, Writing – review & editing. ZZ: Software, Writing – review & editing. CS: Supervision, Writing – review & editing.
